# Human blood neutrophils generate ROS through FcγR-signaling to mediate protection against febrile *P. falciparum* malaria

**DOI:** 10.1038/s42003-023-05118-0

**Published:** 2023-07-18

**Authors:** Ebenezer Addo Ofori, Asier Garcia-Senosiain, Mohammad Naghizadeh, Ikhlaq Hussain Kana, Morten Hanefeld Dziegiel, Bright Adu, Subhash Singh, Michael Theisen

**Affiliations:** 1 Department for Congenital Disorders, Statens Serum Institut, Copenhagen, Denmark; 2 Centre for Medical Parasitology at Department of Immunology and Microbiology, University of Copenhagen, Copenhagen, Denmark; 3 Blood Bank KI 2034, Department of Clinical Immunology, Copenhagen University Hospital, Copenhagen, Denmark; 4 Department of Clinical Medicine, University of Copenhagen, Copenhagen, Denmark; 5 Noguchi Memorial Institute for Medical Research, College of Health Sciences, University of Ghana, Legon, Accra Ghana; 6 ICMR-Regional Medical Research Centre, Bhubaneswar, Odisha India

**Keywords:** Malaria, Antimicrobial responses, Parasite host response, Monocytes and macrophages, Neutrophils

## Abstract

Blood phagocytes, such as neutrophils and monocytes, generate reactive oxygen species (ROS) as a part of host defense response against infections. We investigated the mechanism of Fcγ-Receptor (FcγR) mediated ROS production in these cells to understand how they contribute to anti-malarial immunity. *Plasmodium falciparum* merozoites opsonized with naturally occurring IgG triggered both intracellular and extracellular ROS generation in blood phagocytes, with neutrophils being the main contributors. Using specific inhibitors, we show that both FcγRIIIB and FcγRIIA acted synergistically to induce ROS production in neutrophils, and that NADPH oxidase 2 and the PI3K intracellular signal transduction pathway were involved in this process. High levels of neutrophil ROS were also associated with protection against febrile malaria in two geographically diverse malaria endemic regions from Ghana and India, stressing the importance of the cooperation between anti-malarial IgG and neutrophils in triggering ROS-mediated parasite killing as a mechanism for naturally acquired immunity against malaria.

## Abstract (web summary)

FcγR-mediated signaling by cytophilic IgG against Plasmodium falciparum merozoites triggers ROS generation predominantly in neutrophils as a mechanism contributing to naturally acquired immunity against malaria in endemic regions in Ghana and India.

## Introduction

*Plasmodium falciparum* malaria remains one of the greatest public health challenges, with an estimated 247 million new cases and 619,000 malaria-related deaths worldwide in 2021^1^. The parasite is relatively hidden from the immune system during its asexual stage development, which is associated with malaria symptoms and mortality. On the other hand, merozoites released upon completion of the intraerythrocytic development, are highly vulnerable when they transit from one host cell to another and thus become potential targets of host defense mechanisms (reviewed in refs. ^2–4^). Immunoglobulin (Ig) G antibodies constitute one of the main defenses against blood-stage malaria parasites^5,6^. These antibodies can exert their anti-parasite effects in cooperation with blood leukocytes (e.g., monocytes and neutrophils) through antibody-dependent cellular mechanisms^7–11^.

Both neutrophils and monocytes constitute the dominant phagocyte population (about 55 to 75% of all blood leukocytes), and being the first line of innate defense and effectors of adaptive immunity, both share many features, but possess distinct morphological and functional properties^12,13^. For instance, the metabolic burst activity of monocytes is less strong, but their capacity to kill microorganisms is more diverse compared to neutrophils^14^. Several studies have suggested that both neutrophils and monocytes may eliminate malaria parasites through phagocytosis^9,10,15–17^ and through the production of soluble factors with anti-parasite effects^16,18–20^. Each of these effector functions requires the engagement of surface exposed FcγR by anti-parasite IgG antibodies^10,15,16^. In continuation with our efforts to dissect the contribution of different IgG-mediated cellular responses in protection against malaria, we studied the role of FcγR-triggered generation of ROS in protection against malaria.

Cross-linking of FcγRs on neutrophils and monocytes can trigger the generation of ROS through activation of the Src family kinases, Syk recruitment to the signaling complex, and PI3K activation, which plays an important role in nicotinamide adenine dinucleotide phosphate (NADPH) oxidase (NOX2) activation^21,22^. This leads to the production of large amounts of superoxide anion, leading to the generation of hypochlorous acid (HOCl) in a reaction catalyzed by myeloperoxidase (MPO)^23^. As NOX2 is expressed on both the phagosomal and the plasma membrane, phagocytes can release ROS both intracellularly into the phagosome and into the extracellular space^24–26^.

Here, we used peripheral blood leukocytes (PBLs) to study ROS production intracellularly and extracellularly using freshly purified *P. falciparum* merozoites and naturally occurring antibodies from samples from well-established longitudinal cohort studies (LCS) performed in Ghana and India.

## Results

### Anti-merozoite antibodies elicit ROS generation in neutrophils and monocytes

**Fig. 1:**
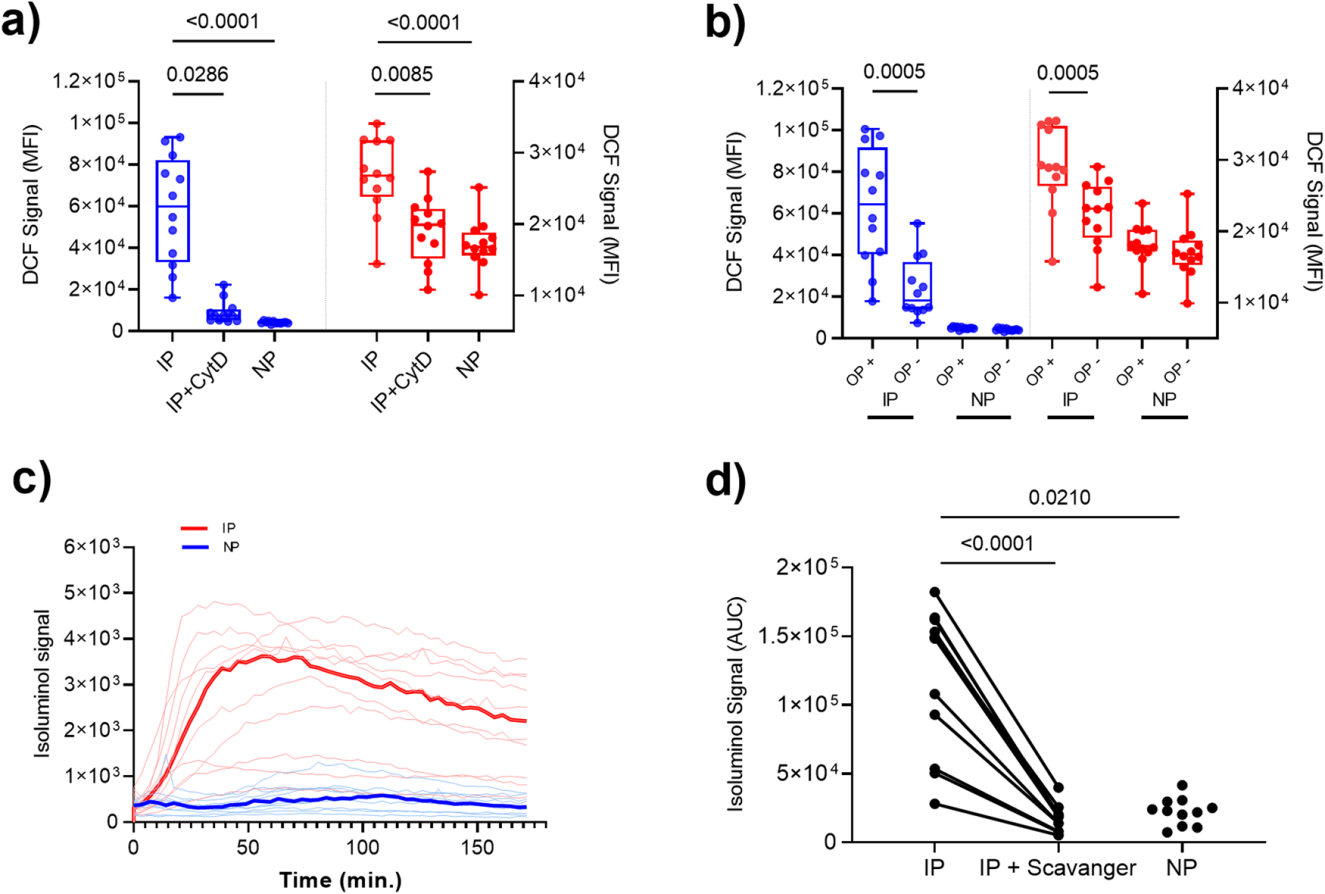
IP-opsonized merozoites stimulate ROS generation by neutrophils and monocytes. **a** Median DCF fluorescence intensity (MFI) of neutrophils (blue) and monocytes (red) after incubation with merozoites opsonized with immune plasma (IP), IP and cytochalasin D (IP + CytD), or non-immune plasma (NP); and **b** phagocytes with engulfed parasite (OP+, detected by EtBr signal) of IP-opsonized merozoites show higher levels of DCF signals compared to those without detectable engulfed parasites (OP−, no EtBr signal). Left y-axes show the MFI of neutrophils, whereas the right y-axes show the MFI of monocytes for the DCF signal. Boxes indicate the median and interquartile range. **c** Kinetics of isoluminol chemiluminescence for peripheral blood leukocytes (PBLs) incubated with merozoites opsonized with IP (red) and NP (blue). Median values are in bold. **d** Area under curve (AUC) of isoluminol chemiluminescence for PBLs incubated with merozoites opsonized with IP, NP, and IP plus the ROS scavengers (catalase and superoxide dismutase) (IP + Scavenger). Values shown are from a single independent experiment (*n* = 12) (i.e., single IP and single NP sample were tested using the same batch of merozoites with PBLs from 12 donors in a single assay). *P* values for panel **b** were determined by Wilcoxon signed-rank test, whereas for panels (**a**, **d**) were determined by a Friedman test and Dunn’s multiple comparisons test.

To investigate ROS generation in response to *P. falciparum* merozoites, we used PBLs from whole blood samples to ensure that the phagocytes were present in their natural physiological proportions and to maximize their cellular integrity. Each experiment used blood from malaria-naïve blood donors (*n* = 12) and merozoites obtained from a single batch of opsonization with single immune plasma, [IP] and single non-immune plasma, [NP] samples, respectively, to minimize assay variability. First, we used the cell-permeable, broadly reacting redox-sensitive fluorescent probe (DCFH_2_-DA) for real-time monitoring and quantification of the intracellular ROS activity ^27^. Both neutrophils and monocytes generated high amounts of ROS when exposed to merozoites opsonized with decomplemented malarial IgG antibodies at a dilution of 1:100, as detected by DCF fluorescence quantified by FACS (Fig. 1a). In the presence of IP, the DCF signal in neutrophils (median MFI = 59,913) was higher as compared to that in monocytes (median MFI = 26,769). Neutrophils and monocytes stimulated with merozoites opsonized with non-immune plasma (NP) displayed 14.7 (95% CI, 8.10–17.50) and 1.6 (95% CI 1.40–1.70) -fold lower DCF signal compared to that of IP-opsonized merozoites respectively, demonstrating that DCF fluorescence induced by ROS is malaria-specific with better signal-to-noise ratio observed for neutrophils as compared to the monocytes. The DCF signal could be abolished by pretreatment of cells with cytochalasin D (Cyt D), suggesting that intracellular ROS generation is related to merozoite-phagocytosis (Fig. 1a). Additionally, we found that within the phagocytes exposed to IP-opsonized merozoites, those with detectable phagocytosed merozoites (indicated by EtBr signal) contained higher levels of intracellular ROS confirming that the DCF signal is linked to opsonic phagocytosis (Fig. 1b).

Next, we used time-lapse confocal microscopy to investigate the relationship between phagocytosis and ROS production. PBLs were incubated with EtBr-stained IP-opsonized merozoites and ROS production was monitored with DCFH_2_-DA. Following phagocytosis, there was a rapid increase in the DCF signal, confirming that these processes are connected (Supplementary Movie 1).

We used membrane-impermeable isoluminol for the detection of extracellular ROS. PBLs produced much higher isoluminol-enhanced ROS signal when exposed to merozoites opsonized with IP compared to NP, demonstrating that extracellular ROS is malaria-specific (Fig. 1c). Addition of superoxide dismutase (SOD) and catalase (CAT) reduced the isoluminol-enhanced ROS signal by 8.1 (95% CI 5.40–8.30)-fold (Fig. 1d). Since SOD and CAT do not penetrate the cell membrane, these scavengers selectively deplete the extracellular ROS.

### IP-opsonized merozoite-induced ROS generation by blood phagocytes depends on FcγRs

**Fig. 2:**
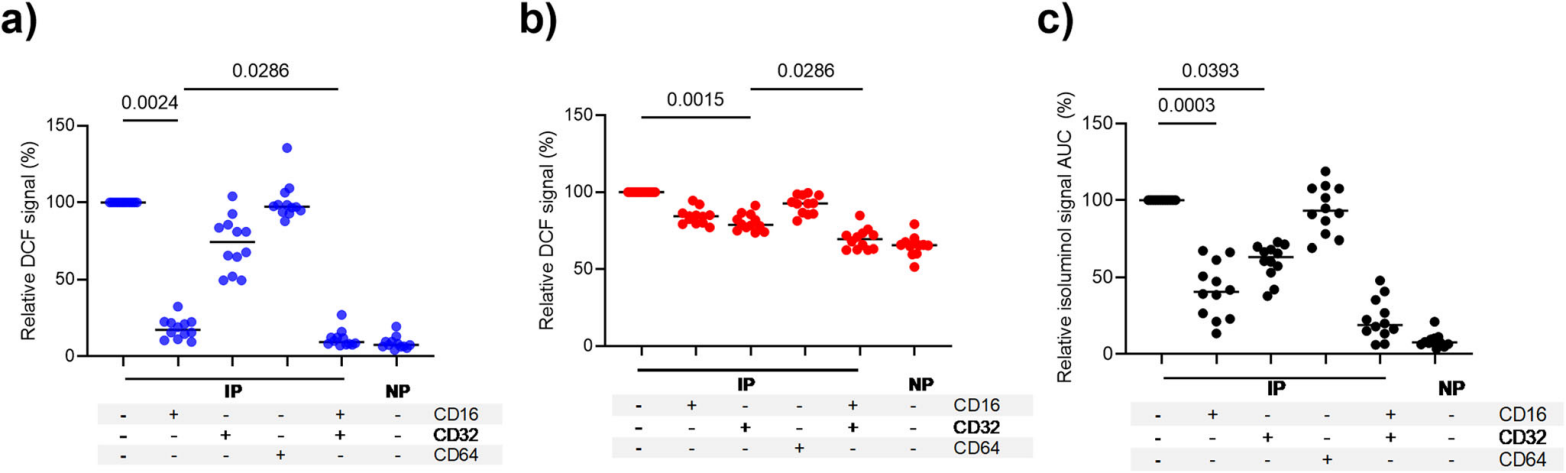
ROS generation after incubation with IP-opsonized merozoites depends on FcγRIII (CD16) in neutrophils and FcγRII(CD32) in monocytes. PBLs treated with 10 μg/ml of antibodies against CD16 (FcγRIII), CD32 (FcγRII), or CD64 (FcγRI) for 30 min were then incubated with IP-opsonized merozoites. Graphs show the relative DCF of neutrophils (**a**) and monocytes (**b**) or relative area under curve (AUC) of isoluminol signal of PBLs (**c**) of specific FcγR blocker using the untreated cells (IP) as reference. Horizontal lines represent median values. P values were determined by the Friedman test and Dunn’s multiple comparisons test. The values shown are from a single independent experiment (*n* = 12).

We used specific monoclonal antibodies (mAbs) to block individual FcγRs, to investigate the role of FcγR-signaling in the generation of ROS. Pre-incubation with anti-CD16 (FcγRIII) antibody led to an 82.7% reduction of intracellular oxidation in neutrophils as detected by the DCF probe (Friedman test, *P* = 0.0024, Fig. 2a). Addition of anti-CD32 (FcγRII) antibody further reduced the remaining DCF signal by another 9.9% compared to anti-CD16 antibody alone (Friedman test, *P* = 0.0286) suggesting that FcγRIIIB and FcγRIIA act synergistically in neutrophils for ROS generation. In contrast, the anti-CD32 antibody alone reduced the generation of ROS in monocytes by 21.4% (Friedman test, *P* < 0.0015, Fig. 2b), demonstrating a significant role of FcγRIIA in ROS generation. The addition of an anti-CD16 antibody further reduced the remaining DCF signal by another 9.2% compared to the anti-CD32 antibody alone (Friedman test, *P* = 0.0286), suggesting that FcγRIIA and FcγRIIIA (as FcγRIIIB expression is known to be absent in monocytes) act synergistically in monocytes for ROS generation. We observed that the combined effect of the anti-CD32 and CD16 antibodies reduced the monocyte DCF signal to that of the background level obtained with NP (Fig. 2b).

Generation of extracellular ROS by PBLs, as detected by isoluminol, was significantly inhibited by the anti-CD16 antibody (59%; Friedman test, *P* = 0.0003) and anti-CD32 antibody (40%; Friedman test, *P* = 0.0393) individually (Fig. 2c), and synergistically by a mixture of both antibodies (78%; Friedman test, *P* < 0.0001) indicating that both FcγRIIIA/B and FcγRIIA are involved in generation of extracellular ROS. Moreover, the anti-CD64 antibody neither inhibited the DCF nor the isoluminol signals, indicating that ROS generated by phagocytes upon opsonic phagocytosis of merozoites does not involve FcγRI.

### ROS generation by IP-opsonized merozoites in blood phagocytes depends on NOX2

**Fig. 3:**
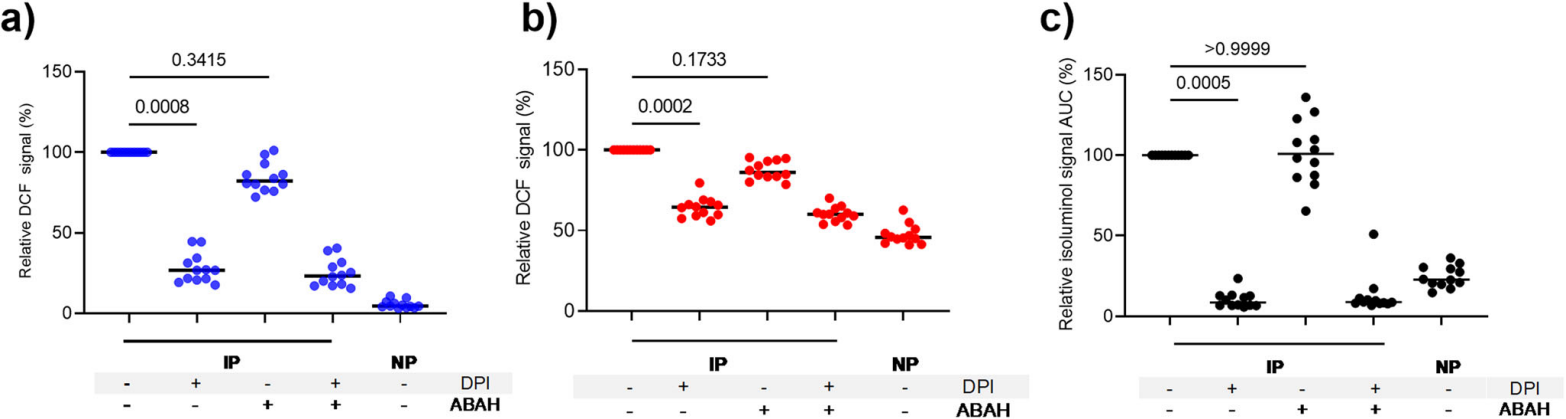
ROS generation by IP-opsonized merozoites in blood phagocytes depends on nicotinamide adenine dinucleotide phosphate (NADPH) oxidase (NOX2). PBLs were incubated with immune plasma (IP) or non-immune plasma (NP) plus 2.5 µM diphenyleneiodonium (DPI, an inhibitor of NOX2) and/or 500 µM 4-aminobenzoic acid hydrazide (ABAH, an inhibitor of myeloperoxidase (MPO)). Graphs show the relative DCF or isoluminol signal of neutrophils (**a**), monocytes (**b**), and PBLs (**c**) using the untreated cells (IP) as reference. Horizontal lines represent median values. *P* values were determined by the Friedman test and Dunn’s multiple comparisons test. The values shown are from a single independent experiment (*n* = 12).

For understanding the mechanism of FcγR signaling induced by IP-opsonized merozoites for the generation of ROS by PBLs, we used diphenyleneiodoium (DPI), a commonly used inhibitor of NOX2^28^, and compared it to DPI-mediated inhibition of NOX2 activation by phorbol 12-myristate 13-acetate (PMA), a known NOX2 activator through protein kinase C signaling^27^. PMA stimulated high amounts of ROS generation in neutrophils (median MFI = 395,646) and monocytes (median MFI = 100,310), as detected by the DCF probe. As expected, DPI abrogated the PMA-enhanced DCF signal in neutrophils and monocytes (Supplementary Fig. 2a, b). Similarly, the addition of DPI reduced the DCF signal in neutrophils by 72% (Friedman test, *P* = 0.0008) and in monocytes by 36% (Friedman test, *P* = 0.0002) after stimulation with IP-opsonized merozoites, demonstrating a role for NOX2 in intracellular ROS production by both phagocytes through FcγR signaling (Fig. 3a, b). The superoxide anion generated by NOX2 is likely to generate the more reactive HOCl by MPO in combination with H_2_O_2_ and chloride^29^. Pre-incubation of the PBLs with the MPO inhibitor 4-aminobenzoic acid hydrazide (ABAH) before adding opsonized merozoites reduced the DCF signal in neutrophils by 17.8% and in monocytes by 13.9% (Fig. 3a, b). However, these reductions did not reach statistical significance.

The addition of DPI to PBLs reduced the isoluminol signal by 91.5% (Friedman test, *P* = 0.0005). However, no reduction was seen after the addition of ABAH (Friedman test, *P* > 0.9999) and combined use of both DPI and ABAH did not cause any further reduction suggesting that extracellular ROS is likely generated through assembly / activation of NOX2 at the plasma membrane^30^. Taken together, these data suggest that IP-opsonised merozoites stimulate FcγR-driven assembly of the NOX2 system either within the phagosomal or on the plasma membrane impacting ROS generation both intracellularly as well as in the extracellular vicinity. Though our experimental design precludes direct assessment of the exact cell type contributing to the generation of extracellular ROS, striking similarities in the patterns observed between Fig. 2a, c, and between Fig. 3a, c, strongly suggest neutrophils to be the dominant cell population contributing to the generation of extracellular ROS.

### PI3K mediated signaling involved in ROS generation stimulated by IP-opsonized merozoites

**Fig. 4:**
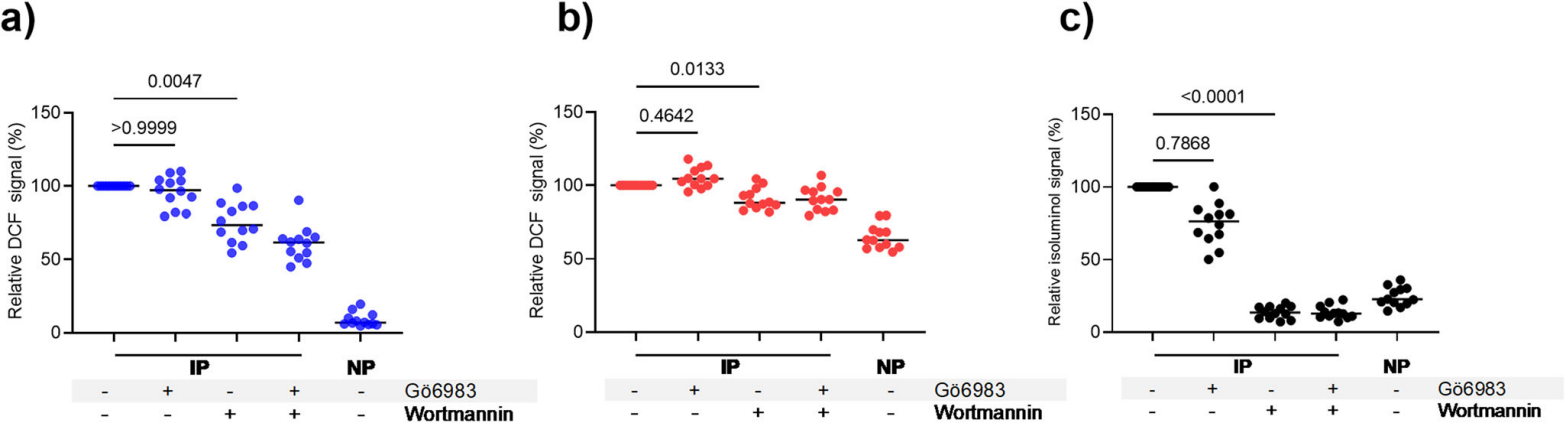
IP-opsonized ROS generation in blood phagocytes depends on the phosphoinositide 3-kinase signaling pathway. PBLs were incubated with immune plasma (IP) or non-immune plasma (NP) plus 250 nM Gö6983 (an inhibitor of protein kinase C) and/or 100 nM wortmannin (an inhibitor of phosphoinositide 3-kinase). Graphs show the relative DCF or relative area under curve (AUC) of isoluminol chemiluminescence signal of neutrophils (**a**), monocytes (**b**), and PBLs (**c**) using the untreated cells (IP) as reference. Horizontal lines represent median values. *P* values were determined by the Friedman test and Dunn’s multiple comparisons test. The values shown are from a single independent experiment (*n* = 12).

We used specific protein kinase inhibitors of PKC and phosphoinositide 3-kinase (PI3K)^27,31^, both known to be involved in NOX2 activation, to dissect the exact mechanism of ROS generation by PBLs upon stimulation with IP-opsonised merozoites. The addition of the PI3K inhibitor wortmannin before adding IP-opsonized merozoites to PBLs significantly reduced the DCF signal in neutrophils (Friedman test, *P* = 0.0047) and monocytes (Friedman test, *P* = 0.0133) as well as the isoluminol signals (Friedman test, *P* < 0.0001) indicating inhibition of ROS generation (Fig. 4a–c). In contrast, the addition of the PKC inhibitor, Gö6983, did not inhibit intracellular (Fig. 4a, b) or extracellular ROS (Fig. 4c) generated through FcγR signaling. As expected, Gö6983 abolished PMA-induced oxidation, while wortmannin had no such effect (Supplementary Fig. 2c, d). Thus, PI3K activation is specifically involved in FcγR-signaling, to stimulate NOX2 activation leading to both intracellular and extracellular ROS generation upon stimulation of PBLs with IP-opsonized merozoites. Treatment with wortmannin reduced the generation of extracellular ROS to near basal levels, whereas it resulted in lower reduction of the intracellular ROS, indicating differential NOX2 activation at the phagosomal and plasma membranes.

Since we found that the generation of intracellular ROS in response to IP-opsonised merozoites was dependent on OP (Fig. 1b), we sought to investigate whether the same FcγR-signaling pathway affected both the IP-mediated phagocytosis process as well as the resulting ROS generation. For this, we assessed the efficiency of phagocytosis of the IP-opsonised merozoites by PBLs in the presence of wortmannin or Gö6983, as indicated by EtBr signals (used to label the merozoite DNA) in the phagocytes. Interestingly, neither of the two kinase inhibitors alone nor in combination had any significant effect on the efficiency of phagocytosis of the IP-opsonised merozoites, indicating that the FcγR-mediated phagocytosis of the opsonized merozoites per se did not depend on PI3K / PKC signaling, which is distinct from the downstream PI3K-signaling events involved in FcγR-mediated generation of ROS in blood phagocytes (Supplementary Fig. 3).

### ROS generated in blood phagocytes by IP-opsonized merozoites is associated with protection against febrile malaria

**Fig. 5:**
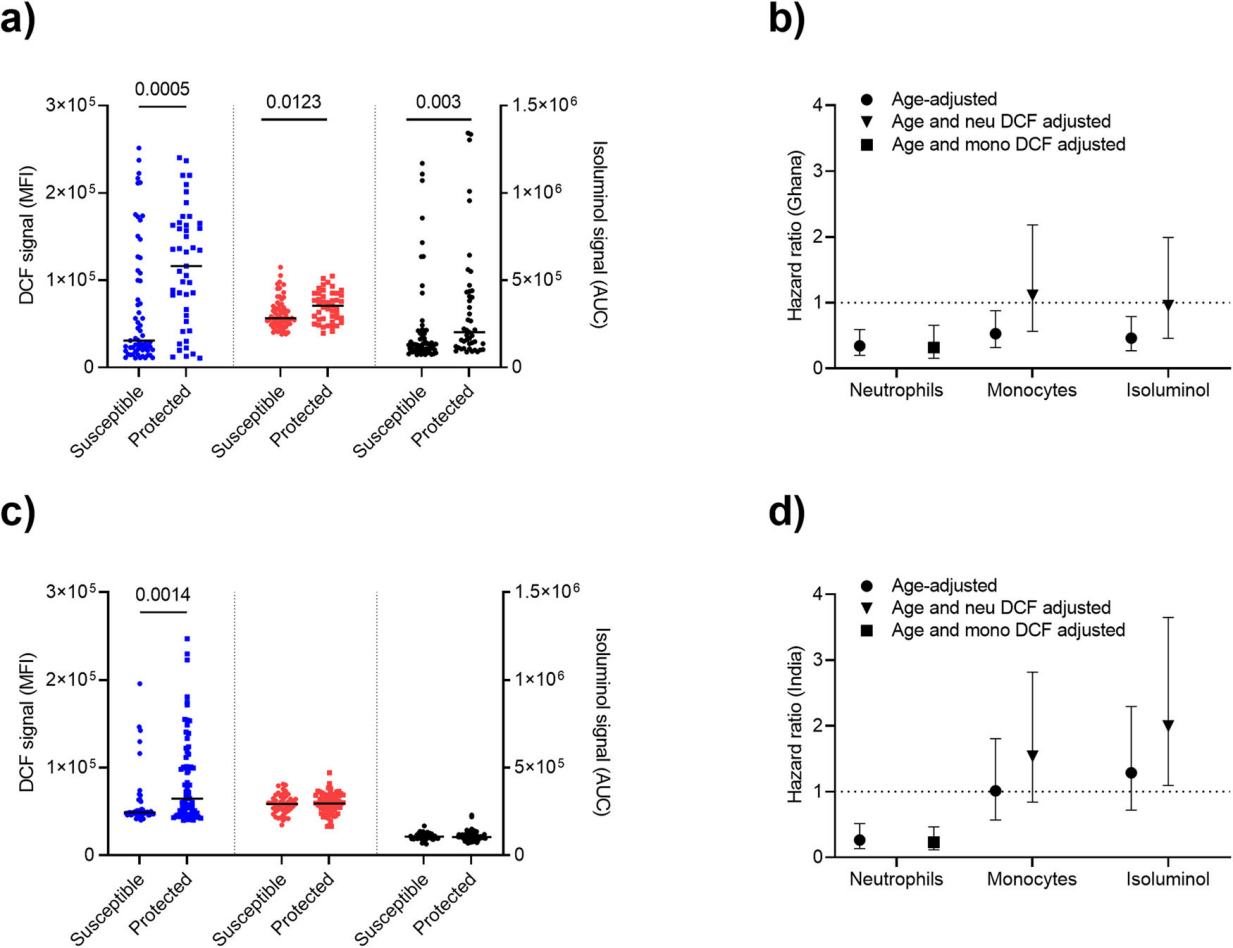
ROS generated in blood phagocytes by IP-opsonized merozoites is associated with protection from febrile malaria. PBLs incubated with merozoites opsonized with plasma samples (108 Ghanaian and 121 Indian cohorts) were analysed for DCF signal and isoluminol signal quantification. Study participants were classified into susceptible and protected individuals (Ghanaian: *n* = 63 and 45, respectively and Indian: *n* = 48 and 73, respectively) based on their febrile malaria status. The DCF signal of susceptible and protected Ghanaian and Indian cohorts (**a**, **c**, respectively) were compared in neutrophils (blue) and monocytes (red). Moreover, isoluminol signal (AUC) in PBLs was compared between susceptible and protected cohorts (**a**, **c**, black). Left y-axis (**a**, **c**) shows the median fluorescence intensity (MFI) of neutrophils and monocytes DCF signal. Right y-axis (**a**, **c**) shows the area under the curve (AUC) of the PBLs isoluminol signal. Ghanaian and Indian cohorts (**b**, **d**, respectively) were categorized into two equal groups based on the median DCF signal, and to calculate the risk of suffering from febrile malaria during the follow-up period, the Cox-regression model was used to compare the high group with the low group (reference group). Values represent age-adjusted (circles), age-plus monocytes DCF (mono)-adjusted (square), and age-plus neutrophils DCF (neu)-adjusted (triangles) hazard ratios at 95% confidence intervals. *P* values were determined by Mann–Whitney tests.

Having demonstrated that IP-opsonized merozoites stimulate PBLs to generate ROS, we determined whether such responses are associated with immunity against clinical malaria using archived plasma samples from a longitudinal cohort survey (LCS) consisting of 108 Ghanaian children who were considered “definitively” exposed (i.e., children who had parasitemia at one or more of the monthly blood slides during the study) and who had completed follow-up^32^. ROS in neutrophils (Mann–Whitney test; *P* = 0.0005) and monocytes (Mann–Whitney test; *P* = 0.0123), as well as extracellular ROS (Mann–Whitney test; *P* = 0.0030), were generated at significantly higher levels by plasma samples from “protected” compared to the non-protected children (Fig. 5a). Children were categorized into groups with high and low DCF- or isoluminol signals based on the median in order to examine the risk of febrile malaria for each group using Cox-regression models to calculate Hazards ratios (HRs). After adjustment of age, children with high levels of DCF signal (age-adjusted [aHR] = 0.34; 95% CI = 0.19–0.59; *P* < 0.0001 for neutrophils and aHR = 0.53; 95% CI = 0.32–0.88; *P* = 0.0139 for monocytes) and isoluminol signal ([aHR] = 0.46; 95% CI = 0.27–0.79; *P* = 0.0046) had significantly higher probability of remaining free of febrile malaria compared to those with low-level responses (Fig. 5b). Since the DCF signal of neutrophils was highly correlated to the isoluminol signal (Pearson *r* = 0.6700, *P* < 0.0001) (Supplementary Fig. 4a) and the monocytes (Pearson *r* = 0.8558, *P* < 0.0001) (Supplementary Fig. 4b), Cox-regression models were also adjusted for neutrophil ROS as a confounder. The magnitude of protective associations of both monocyte ROS and extracellular ROS were significantly influenced by neutrophil ROS (Fig. 5b).

Next, we investigated samples and clinical data from an LCS conducted in India^33^. Intracellular ROS generated by IgG from Indian plasma samples in neutrophils ([aHR] = 0.26; 95%CI 0.13–0.52; *P* = 0.0001) but not in monocytes ([aHR] = 1.01; 95% CI = 0.57–1.80; *P* = 0.965) was strongly associated with protection against febrile malaria (Fig. 5c, d). Extracellular ROS was not associated with protection ([aHR] = 1.28; 95%CI 0.72–2.29; *P* = 0.3962) from clinical malaria in this Indian cohort (Fig. 5d). Cox-regression models were adjusted for neutrophil ROS as a confounder as neutrophils DCF signal highly correlated to the isoluminol signal (Pearson *r* = 0.5115, *P* < 0.0001) (Supplementary Fig. 4c) and moderately correlated to monocytes (Pearson *r* = 0.4413, *P* < 0.0001) (Supplementary Fig. 4d).

To further strengthen the contribution of neutrophils ROS to malaria immunity, time to the first episode in the two LCS were analyzed by logistic regression models that included age and monocyte ROS or extracellular ROS (model 1) were compared to a second logistic regression model (model 2), which was fitted for neutrophil ROS in addition to the variables in model 1 (Table 1). The results confirmed that neutrophil ROS had significantly impacted the protective associations of monocyte ROS and extracellular ROS in Ghana (*P*_LRT_ = 0.00296) and India (*P*_LRT_ < 0.0001). Thus, neutrophil ROS is the main predictor of protection against febrile malaria across diverse geographic malaria-endemic regions in Ghana and India.

**Table 1:** ROS responses associated with protection against malaria.

Functional activity	Model 1	Model 2	LRT *P* value
	OR (95% CI)		
Ghanaian LCS			
Neutrophil ROS	NA	0.16 (0.04–0.55)	**0.00296**
Monocyte ROS	0.38 (0.16–0.83)	1.31 (0.40–5.13)	
Neutrophil ROS	NA	0.15 (0.03–0.53)	**0.0034**
Isoluminol level	0.35 (0.15–0.80)	1.51 (0.40–7.39)	
Indian LCS			
Neutrophil ROS	NA	0.15 (0.06–0.38)	**<0.0001**
Monocyte ROS	0.98 (0.46–2.11)	1.73 (0.73–4.38)	
Neutrophil ROS	NA	0.13 (0.05–0.32)	**<0.0001**
Isoluminol level	1.47 (0.67–3.27)	2.85 (1.14–7.84)	

The table shows the *P* value obtained from the LRT, which compares a reduced logistic regression model (model 1) with a full logistic regression model (model 2). Model 1 was fitted for age groups (1–5 or 6–12 years in Ghana; ≤10, 11–15, or ≥16 years in India) and monocyte ROS or extracellular ROS, whereas model 2 was fitted for neutrophil ROS in addition, to the variables in model 1. Significant LRT *P* values are in bold font.

*CI* confidence interval, *LRT* likelihood ratio test, *OR* odds ratio, *ROS* reactive oxygen species.

### Correlation between ROS-levels and opsonic phagocytosis (OP) activity

**Fig. 6:**
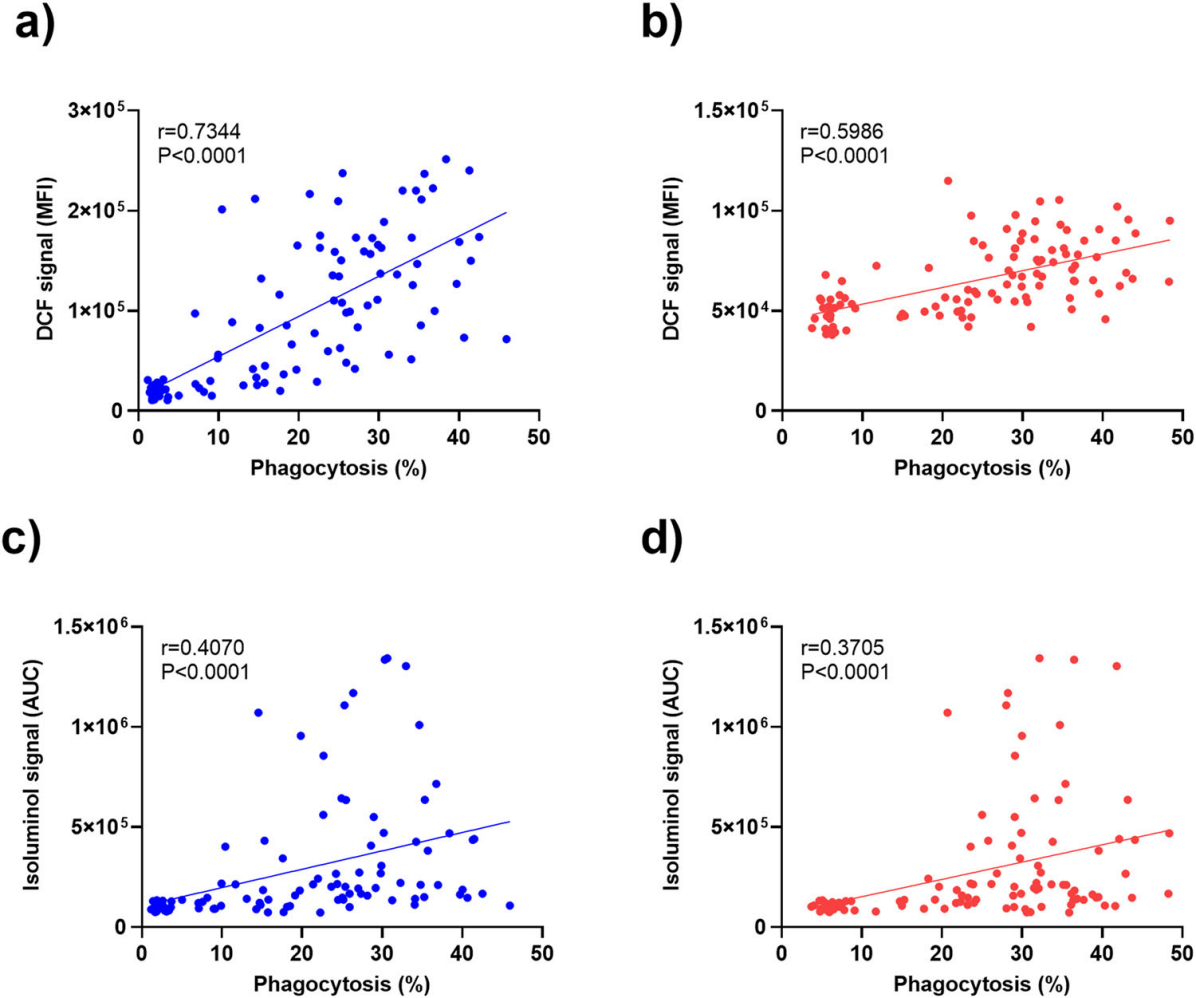
Correlation between opsonic phagocytosis of IP-opsonized merozoites and ROS generation in blood phagocytes. Scatterplots with linear regression lines show the relationship between intracellular ROS production and opsonic phagocytosis in neutrophils (**a**) and monocytes (**b**). The production of extracellular ROS and opsonic phagocytosis in neutrophils and monocytes are depicted in panels **c**, **d**, respectively. Pearson’s correlation coefficient (*r*) and corresponding *P* values are shown in each plot (*n* = 108).

In a previously reported study, the OP activity of the same IgG preparations from Ghanaian samples were used in the present study was associated with protection from febrile malaria^10^. Since the ROS and OP bioassays share fundamental similarities, we investigated possible relationships between these two assays. Strong correlations were observed between neutrophil ROS and OP (Pearson *r* = 0.7344, *P* < 0.0001) and monocyte ROS and OP (Pearson *r* = 0.5986, *P* < 0.0001). In contrast, associations between isoluminol ROS and neutrophil OP (Pearson *r* = 0.4070, *P* < 0.0001) and monocyte OP (Pearson *r* = 0.3705, *P* < 0.0001) were less strong and showed frequent samples that elicited high responses in one assay and low in the other (Fig. 6).

### Effect of ROS responses on antibody-mediated protection against febrile malaria

To determine the contribution of specific ROS activities to malaria immunity acquired through anti-merozoite IgG antibodies, LRTs were performed as follows. A logistic regression model that included age and merozoite-phagocytosis (model 1) was compared to another logistic regression model (model 2), which was fitted for ROS in addition to the variables in model 1. It was observed that the ROS activity of neutrophils, but not monocytes, had a significant influence on the probability of developing febrile malaria in Ghana (OR = 0.12, 95% CI = 0.02–0.041; *P* = 0.0006) and India (OR = 0.31, 95% CI = 0.11–0.81; *P* = 0.0177) suggesting that ROS contributes, at least in part, to the antibody-associated protection against febrile malaria observed here (Table 2).

**Table 2:** ROS responses and merozoite-phagocytosis (OP) associated protection against malaria.

	Model 1	Model 2	
Functional activity	OR (95% CI)	OR (95% CI)	LRT *P* value
Ghanaian LCS			
Neutrophil OP	0.46 (0.20–1.00)	2.04 (0.59–9.55)	**0.0006**
Neutrophil ROS	NA	0.12 (0.02–0.41)	
Monocyte OP	0.41 (0.18–0.90)	0.64 (0.22–1.88)	0.2082
Monocyte ROS	NA	0.51 (0.17–1.46)	
Indian LCS			
Neutrophil OP	0.22 (0.09–0.49)	0.41 (0.15–1.09)	**0.0177**
Neutrophil ROS	NA	0.31 (0.11–0.81)	
Monocyte OP	0.53 (0.25–1.14)	0.52 (0.23–1.13)	0.7407
Monocyte ROS	NA	1.14 (0.52–2.54)	

The table shows the *P* value obtained from the LRT, which compares a logistic regression model (model 1) that includes merozoite-phagocytosis activity categorized into two groups divided by the median value as indicated and age groups (1–5 or 6–12 years) with a second logistic regression model (model 2) that also includes ROS-levels categorized into two groups by the median value in addition to the variables in model 1. The ORs shown refer to the indicated functional activities. Significant LRT *P* values are in bold font.

*CI* confidence interval, *LRT* likelihood ratio test, *OP* opsonic phagocytosis, *OR* odds ratio.

## Discussion

Cytophilc IgG against malaria antigens play a vital role in naturally acquired immunity (NAI) against malaria^7,34,35^ by mediating a variety of anti-malaria effector functions through interactions with the FcγRs expressed on the leukocyte surface^15,34,36,37^. These effector functions include stimulation of distinct leukocyte subsets to secrete soluble cytokines^35,38^ for arresting the intraerythrocytic development of the malaria parasites or enhancing their elimination through OP by blood phagocytes^10,39^. Here we found that OP leads to the generation of both intracellular and extracellular ROS predominantly in neutrophils, but also in monocytes. Moreover, the ability of the plasma samples to stimulate neutrophil ROS generation is strongly associated with protection against febrile malaria, indicating functional specialization amongst different leukocyte lineages. While the use of PBLs to study ROS generation is attractive from an experimental point of view, it also possessed some inherent challenges in the neutrophil and monocyte populations. Firstly, due to the characteristic fragility of neutrophils, an experiment must utilize samples collected shortly (2–4 h) before use. Secondly, considering the relatively lower signal-to-noise ratio in the monocyte DCF signal, it is critical to minimize inter-assay variability by adopting a design that allows testing of all samples in a single experiment. The higher basal levels of intracellular ROS might be related to the greater content of respiratory active mitochondria in monocytes compared to neutrophils^40^. Irrespective of this physiological difference, we consistently observed around a 1.6-fold difference between IP and NP in monocyte DCF signals between experiments suggesting that monocyte intracellular ROS signals are measured accurately in the assay used here. Thus, by adopting the present procedure, we have limited sample-to-sample variability by harmonizing cell integrity, biochemical and physiological activities, which are likely to degenerate over time.

The differences in expression of the FcγR types and the variations in their binding affinity to the structural heterogeneity of the interacting Fc domains of the opsonizing antibodies^41^, suggests another set of differences in effector functions elicited in the neutrophil and monocyte cell populations. Neutrophils and monocytes both express FcγRIIA, with a subset of non-classical monocytes additionally expressing FcγRIIIA^42^, while FcγRIIIB expression is exclusive to neutrophils^43^. The high density of FcγRIIIB (⁓1.4 × 10^6^ receptors per cell) on neutrophils may cause extensive receptor clustering at the membrane, leading to amplified activation and ROS generation^41^. In neutrophils, FcγRIIIB appears to play a dominant role, with FcγRIIA providing a secondary and synergistic signal for OP and ROS generation in response to IgG opsonized merozoite. The monocytes, in contrast, appear to primarily depend on FcγRIIA-mediated activation signaling^10^ with FcγRIIIA likely to play a small role (in ROS generation but not in OP), indicating that distinct FcγR subsets are involved in eliciting OP and signaling for ROS generation in these blood phagocyte lineages. Signaling through FcγRIIIB, as it lacks its own transmembrane domain, is understood to occur through associations with FcγRIIA^44^ and may mediate a rise in cytosolic Ca^2+^ through mechanisms distinct from that of FcγRIIA activation alone, thereby leading to ROS generation upon activation with IgG opsonized merozoites^20^.

Results from this study demonstrate that treatment of PBLs with a pan-PI3K inhibitor, wortmannin, reduced the generation of extracellular ROS to basal levels and resulted in a consistent and significant reduction in intracellular ROS generation. Wortmannin treatment has been reported to block essential membrane subunits of NOX2^45,46^ and some of these are understood to regulate differential activation of NOX2 between the respective phagosomal and plasma membranes^47^. Whether differential activation of NOX2 explains the lower reduction in intracellular compared to the extracellular ROS generation or other signaling pathways contribute to intracellular ROS generation remains to be investigated. Interestingly, wortmannin did not have any effect on the phagocytic uptake of IgG opsonized merozoites, indicating that mechanisms involved in FcγR dependent phagocytosis are distinct from the downstream PI3K-signaling events involved in the generation of ROS. These results agree with previous reports where the use of PI3K inhibitors (wortmannin or LY294002) also did not affect phagocytosis^38^. Collectively these findings suggest that distinct FcγR signaling mechanisms could be involved in control of OP and ROS generation.

In neutrophils, IgG opsonized merozoites through interactions with the FcγRIIA and FcγRIIIB upon phagocytosis trigger activation of the NOX2 enzyme complex both in the phagosomal as well as plasma membrane, leading to the generation of O2•−, which is readily converted into H_2_O_2_. In the neutrophil phagosomes, H_2_O_2_ is further converted to HOCl and derivatives thereof^48,49^. Inhibition of NOX2 (using DPI) completely abrogated ROS generation by neutrophils and led to a lower though, significant decrease also in monocytes demonstrating the essential involvements of this enzyme in the ROS generation by blood phagocytes. Apparently, the minimum reduction of intracellular ROS levels in monocytes, even after NOX2 inhibition, could result from its high basal levels^40^. Contrary to expectations, inhibition of MPO (using ABAH) had no effect on the DCF signal. It is unclear whether this is due to the specificity and/or sensitivity of the DCFH2-DA dye used for ROS detection in the assays. Regardless of the enzymes involved in generating ROS and downstream halogen species, the role of such compounds in the elimination of malaria parasites requires further investigation.

The role of ROS in providing naturally acquired protection against febrile malaria was investigated using samples and data from two well-established LCS conducted in Ghana and India^33,50^. Results from this study identified neutrophils as the primary blood phagocyte contributing to IgG-mediated ROS generation upon exposure to malaria merozoites. Neutrophil-derived ROS accounted for 66 and 74% reductions in the probability of developing febrile malaria in the Ghanaian and Indian LCS, respectively, after considering the confounding effects of age. This finding is in agreement with a previous study demonstrating that extracellular ROS generated by purified neutrophils was significantly associated with protection from clinical malaria in two distinct areas of Senegal^18^ and underlines the importance of neutrophils in NAI^10,16,18^.

Intracellular ROS has been linked to phagocytosis both in neutrophils and monocytes; thus, we compared individual DCF signals obtained here with previous data on merozoite-OP from the Ghanaian LCS^10^. Neutrophil ROS activity significantly (LRT, *P* = 0.0006) contributed to the protective immunity observed, suggesting that though linked, neutrophil-derived ROS and OP involve distinct operational mechanisms. Extracellular ROS, as detected by isoluminol, was not strongly associated with neutrophil or monocyte OP activities, making the exact role of secreted ROS elusive. Previous studies have demonstrated that neutrophils stimulated with NOX2-activators produced compounds that inhibited the asexual intraerythrocytic *P. falciparum* growth in vitro^19,51^. However, scavengers such as CAT and SOD did not reverse the parasite growth inhibition implying that ROS was not the main mediator of this parasite killing. It may be speculated that ROS acts in an indirect manner through the activation of secretory granule releasing of toxic mediators with anti-parasite activity. The conditions that favor these different immune mechanisms remain to be understood; however, their interconnectedness seems plausible, reinforcing the perception that NAI against malaria is a composite of multiple mechanisms.

To our knowledge, this is the first time that PBLs have been used to monitor ROS generation through FcγR signaling elicited by anti-malarial antibodies and its significance as a strong predictor of protection against clinical malaria has been established in two geographically diverse malaria-endemic regions.

In conclusion, findings from this study have identified neutrophils as the dominant cell type for ROS elicited by anti-merozoite antibodies and have established that a specific FcγR signaling pathway is involved in the generation of ROS predominantly in neutrophils by protective antibodies against malaria. In contrast, monocyte ROS, extracellular ROS, and OP are not significantly associated with clinical protection in either Ghana or India. The relative contribution of ROS as an effector in mediating parasite killing amongst other antibody-triggered leukocyte effector mechanisms remain to be elucidated.

## Methods

### Ethics statement

The Ghanaian longitudinal cohort study was approved (NMIMR-IRB CPN 028/07 −08) by the Institutional Review Board of Noguchi Memorial Institute for Medical Research of the University of Ghana, Accra, Ghana. For the Indian longitudinal cohort study approval (ECR/NIMR//EC/2013//93) was obtained by the Institutional Ethics Committee of the National Institute of Malaria Research, Indian Council of Medical Research, New Delhi, India, and as part of an inter-governmental Indo-Danish research program. Written informed consent was given by study participants or their parents/guardians before sample collection. Ethical approval for Danish blood donor samples was given by the Scientific Ethics Committee of Copenhagen and Frederiksberg, Denmark. Samples from anonymous Danish blood donors (18–60 years of age) obtained for control purposes at Copenhagen University Hospital were used. These individuals are residents of central Copenhagen and provided written consent to have a small portion of their blood stored anonymously and used for research purposes. All data were analyzed anonymously.

### Study populations and study designs

Population characteristics are shown in Supplementary Table 1. Samples and clinical data were from a longitudinal cohort study performed in Asutsuare, Damgbe, West District, Ghana^50,52^. In May of 2008, 798 children aged 12 years and younger were enrolled and venous blood was obtained. The participants were followed up for 42 weeks for active and passive detection of malaria and symptoms. By the end of the study, the cohort was divided into three groups: (1) those susceptible, in which parasitemia was associated with febrile malaria (axillary temperature ≥37.5 °C measured or reported and at least 1 other sign of malaria such as vomiting, diarrhea, or malaise); (2) those apparently protected against clinical manifestation despite parasitemia; and (3) those without detectable parasitemia by microscopy and no clinical manifestations of malaria. Only children in groups 1 and 2 were included in the present study as they were definitively exposed to *P. falciparum* infections. It should be noted that 108 samples were available for the present study compared to the 140 samples previously reported in the same cohort^10^. Individuals were classified either as protected (no cases of febrile malaria during follow-up) or susceptible (one or more cases of febrile malaria).

The Indian study was conducted in Dumargarhi in the state of Jharkhand^33^. A total of 945 individuals aged 1 to 82 were enrolled in May 2014. Blood samples from 386 individuals were obtained in the first cross-sectional survey and participants were followed up actively and passively for malaria detection for 13 months. In the current study, 121 participants who were slide-positive for *P. falciparum* at any of the surveys or who has suffered from a febrile malaria episode were included. These are the same samples used in a previously published study^10^. Febrile malaria was defined as any *P. falciparum* parasitemia confirmed by microscopy of stained thick and thin blood smears plus reported fever or axillary temperature ≥37.5 °C at the time of the visit. Pools of hyperimmune plasma (IP) from malaria-exposed Liberian adults and from non-immune plasma (NP) from Danish blood donors never exposed to malaria served as internal controls^53^.

### Parasite culture and merozoite isolation

*P. falciparum* strain NF54 were cultured as previously described in ref. ^11^. Parasites were cultured in O+ erythrocytes at 4% hematocrit with parasite growth medium (RPMI-1640 supplemented with 25 mM HEPES, 5 g/l AlbuMAX, 4 mM l-glutamine, 0.02 g/l hypoxanthine, and 25 μg/ml gentamicin). The culture was kept at 37 °C in an atmosphere containing 5% O_2_, 5% CO_2_, and 90% N_2_. Parasitemia and developmental stages were monitored with thin blood smears observed by light microscopy. Smears were fixed with methanol and stained with 10% Giemsa for 10 min. Parasites were synchronized by treating with 5% sorbitol for 10 min. After reaching the mature trophozoite/early schizont stage, parasites were purified with a magnetic separation column and cultured further in the parasite growth medium. Mature schizonts were then filtered through a 1.2 μm pore filter to obtain merozoites. Different batches of merozoites were produced for each experiment performed in a day and used at a leukocyte-to-merozoites ratio of 1:4.

### Leukocytes purification

PBLs were isolated from blood samples of healthy Danish donors (*n* = 12). All blood samples were collected within 2–4 h before use. The PBL-containing layer was obtained by centrifugation at 830 × *g* for 25 min at room temperature. Red blood cells remnant were lysed by mixing with 10 parts of lysis buffer (155 mM NH_4_Cl, 10 mM KHCO_3_, and 0.1 mM EDTA; pH 7.4) for 10 min. Cells were then centrifuged at 400 × *g* for 5 min and resuspended in cell medium (RPMI-1640 supplemented with 25 mM HEPES, 10% fetal bovine serum, 4 mM l-glutamine, and 25 μg/ml gentamicin) for intracellular ROS quantification or Krebs–Ringer phosphate buffer (119 mM NaCl, 4.75 mM KCl, 0.420 mM CaCl_2_, 1.19 mM MgSO_4_, 16.6 mM sodium phosphate buffer, and 5.56 mM glucose; pH 7.4) for extracellular ROS quantification. Cells were counted using a hemocytometer and adjusted to 5 × 10^5^ cells/ml and 5 × 10^6^ cells/ml for intracellular and extracellular ROS quantification, respectively.

Neutrophil purification was performed as previously described^10^. In brief, a blood sample from a healthy donor was layered on an isotonic Percoll solution (1.077 g/ml) and centrifuged at 800 × *g* for 25 min. Neutrophils in the lower layer were further purified using the EasySep Human Neutrophil Isolation Kit (StemCell Technologies) following the manufacturer’s instructions after lysis of contaminating red blood cells. Purified neutrophils were adjusted to 5 × 10^5^ cells/ml using a cell medium.

### Intracellular ROS quantification

PBLs were distributed in 96-well U-bottom plates containing 5 × 10^4^ cells in 100 μl of cell medium per well. Then, 50 μl of cell medium with 12 μM of 2’,7’-dichlorodihydrofluorescin diacetate (DCFH_2_-DA, 3 μM final concentration) and 1:200 dilution of surface staining antibodies (1:800 final dilution) were added. The staining antibodies were APC anti-human CD45 (clone HI30; BD Biosciences 555485), BV421 anti-human CD66b (clone G10F5; BD Biosciences 562940), and APC-AF750 anti-human CD14 (clone TuK4; Thermo Fisher Scientific MHCD1427) selective for leukocytes, granulocytes, and monocytes, respectively. Immediately, 50 μl of merozoites resuspended in cell medium and opsonized with immune or non-immune plasma diluted 1:100 was added to each well. After an incubation of 30 min at 37 °C, cells were washed thrice with FACS buffer (PBS with 0.5% BSA + 2 mM EDTA). Samples were quantified with a CytoFLEX S (Beckman Coulter Life Sciences) flow cytometer. DCF signal was determined by measuring the fluorescence using the 525/40 nm detector. Data analysis was performed with Kaluza Analysis Software version 2.1 (Beckman Coulter Life Sciences). The gating strategy used is shown in Supplementary Fig. 1.

### Extracellular ROS quantification

PBLs were dispensed in 96-well white opaque plates with 5 × 10^5^ cells in 170 μl of Krebs–Ringer phosphate buffer per well. Isoluminol was added to a final concentration of 0.04 mg/ml. Then, 30 μl of merozoites resuspended in Krebs–Ringer phosphate buffer and opsonized with immune and non-immune plasma diluted 1:50 were added. Chemiluminescence kinetics were recorded with a TopCount NXT Scintillation and Luminescence Counter or a SpectraMax i3x multi-Mode microplate reader using the SoftMax Pro 7.1 software for data acquisition and analysis.

### Phagocytosis assay (OP)

The process for quantification of merozoite-phagocytosis has been described in detail^10^. Leukocytes were distributed in 96-well U-bottom plates containing 5 × 10^4^ cells in 100 μl of cell medium per well. Ethidium bromide-stained merozoites were resuspended in cell medium, opsonized with immune and non-immune plasma at a 1:100 dilution, and 100 μl were added to each well. After a 30 min incubation at 37 °C, cells were washed thrice with cold FACS buffer. Cells were resuspended in cold FACS buffer containing the following antibodies diluted 1:800: BV421 anti-human CD66b (clone G10F5, BD Biosciences 562940); APC anti-human CD45 (clone HI30, BD Biosciences 555485); and APC-AF750 anti-human CD14 (clone TuK4, Thermo Fisher Scientific MHCD1427) (Supplementary Fig. 1). After a 30 min incubation at 4 °C, cells were washed thrice with cold FACS buffer. Samples were quantified with a CytoFLEX S flow cytometer and analyzed with Kaluza Analysis Software version 2.1. Phagocytosis was measured using the 610/20 nm detector to quantify the ethidium bromide signal.

### Confocal microscopy

Purified neutrophils were incubated with 3 μM DCFH_2_-DA. Merozoites were stained with ethidium bromide and opsonized with immune plasma at a 1:100 dilution. Neutrophils and merozoites were transferred to confocal dishes (VWR734-2903) pretreated with 0.01% poly-l-lysine (Merck P6282). Cells were imaged using an LSM 980 confocal microscope (Zeiss) with a C-Apochromat 40x/1.2 water-immersion objective. Images were recorded at 4 sec intervals during a 4 min timespan.

### Fcγ receptor blocking

Before incubating with merozoites, cells were treated with antibodies against FcγRI (CD64), FcγII (CD32), and FcγIII (CD16), as described previously in ref. ^10^. Cells were treated for 30 min. at 37 °C with 10 μg/ml of anti-human CD64 (clone 10.1, BD Biosciences 555525); anti-human CD32 (clone FLI8.26, BD Biosciences 555447); anti-human CD16 (clone 3G8, BD Biosciences 555404), or a combination of these to block FcγR-I, II, and III, respectively. Merozoites were subsequently added, and the experiments continued as described. Values for treated cells are presented relative to the value of untreated cells.

### Cytochalasin D treatment (CytD)

PBLs in 100 μl of cell medium per well were pretreated for 30 min with 20 µg/ml cytochalasin (CytoD), an inhibitor of actin polymerization, and the experiments continued as described above for the OP assay.

### Enzyme inhibition

Before the addition of opsonized merozoites, cells were incubated for 1 h at 37 °C with 500 μM of MPO inhibitor, 4-aminobenzoic hydrazide (ABAH, Merck A41909); 2.5 μM of NOX2 inhibitor, diphenyleneiodonium chloride (DPI, Merck D2926); 250 nM of PKC inhibitor, Gö 6983 (Merck G1918); 100 nM of P13K inhibitor, Wortmannin (Merck W1628) or a combination of these. After the incubation period, merozoites were added and the assays continued as previously described.

### Statistics and reproducibility

Each experiment included twelve biological replicates. The Mann–Whitney test was used to evaluate differences between two unpaired groups. To assess differences between two groups of paired observations, the Wilcoxon signed-rank test was used. The Friedman test with Dunn’s multiple comparisons test was applied to estimate differences between three or more paired groups. Individuals were stratified into two equal groups (low or high) based on the median DCF or isoluminol signals. The associations between time to the first febrile malaria episode and these categorical variables were analyzed by age-adjusted Cox-regression models. Correlations were assessed with the Pearson correlation coefficient. The significance of the effect of ROS production was assessed by likelihood ratio tests (LRT) comparing a logistic regression model that included age and either monocyte ROS or extracellular ROS as predictors, as well as age and merozoite-phagocytosis with a second model that also included the ROS values. All analyses were performed as two-sided tests. *P* values less than 0.05 were considered significant. Statistical analyses were performed with GraphPad Prism 9 (GraphPad Software, Inc.) and R version 4.1.2 with the survival and lmtest packages^54,55^.

### Reporting summary

Further information on research design is available in the Nature Portfolio Reporting Summary linked to this article.

## Supplementary information

Supplementary Information

Description of Additional Supplementary Files

Supplementary Movie 1

Supplementary Data

Reporting Summary

## Data Availability

The data generated in this study are provided in the Supplementary Data file. Data were also available from the corresponding authors upon request and pending agreement from relevant ethics committees for clinical data.
