# Supplementary material for: Human blood neutrophils generate ROS through FcγR-signaling to mediate protection against febrile P. falciparum malaria
Source: Commun Biol. 2023 Jul 18;6:743. doi: 10.1038/s42003-023-05118-0 (PMC10354059; doi:10.1038/s42003-023-05118-0)
Supplement: Supplementary file 1 — Supplementary Information [file 42003_2023_5118_MOESM1_ESM.pdf]

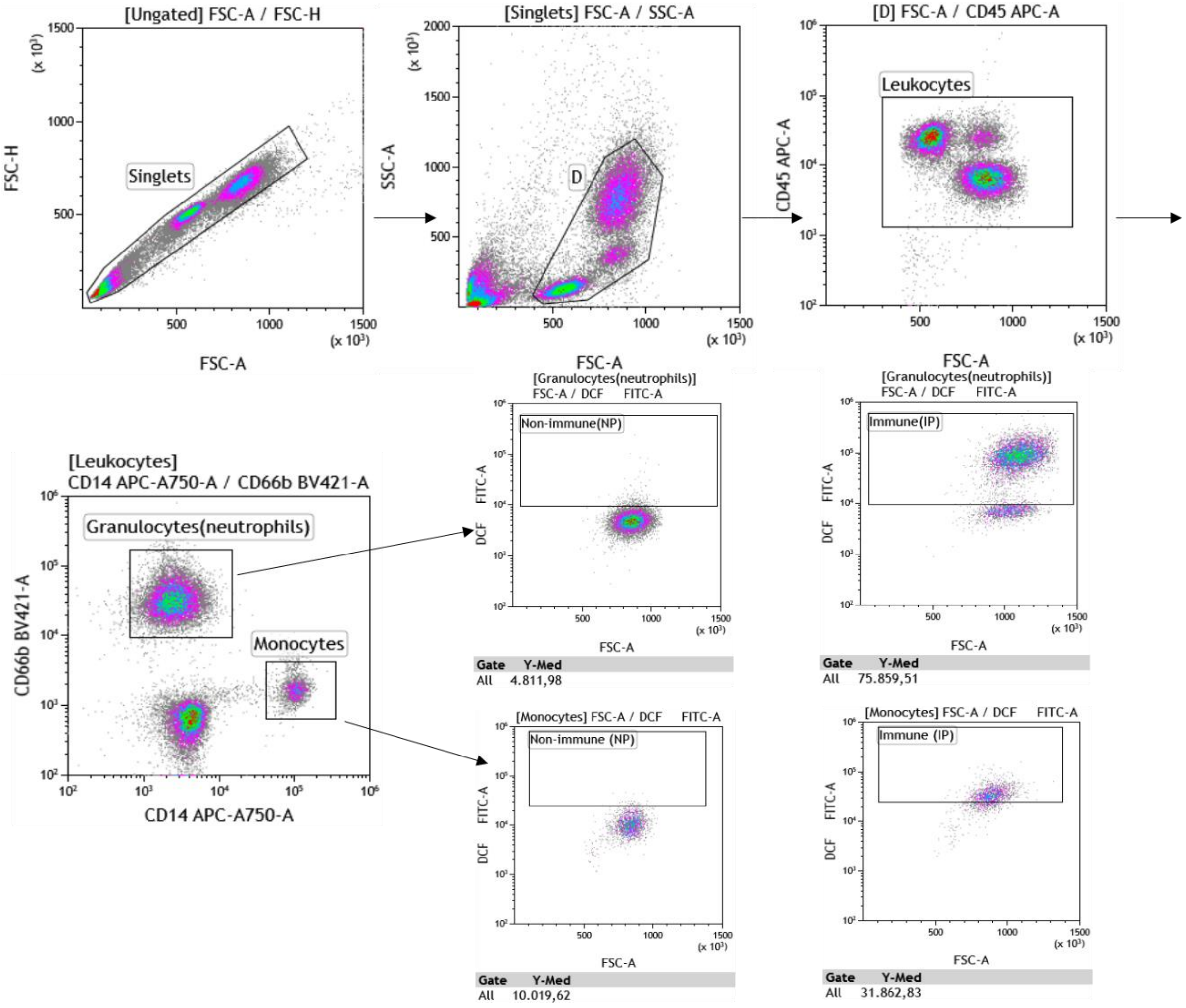

Supplementary Figure 1. Gating strategy for the quantification of reactive oxygen species (ROS) production by neutrophils and monocytes.

Leukocytes were isolated from peripheral blood of healthy donors by centrifugation followed by hypotonic lysis of erythrocytes. Leukocytes were incubated with $DCFH_2$-DA and opsonized merozoites for 30 min at 37°C. The panels show the gating strategy to assess ROS production by neutrophils defined as $CD66b^+/CD14^-$ cells and monocytes defined as $CD14^+/CD66b^-$ cells. DCF signal was measured in the FITC channel (525/40 nm detector).

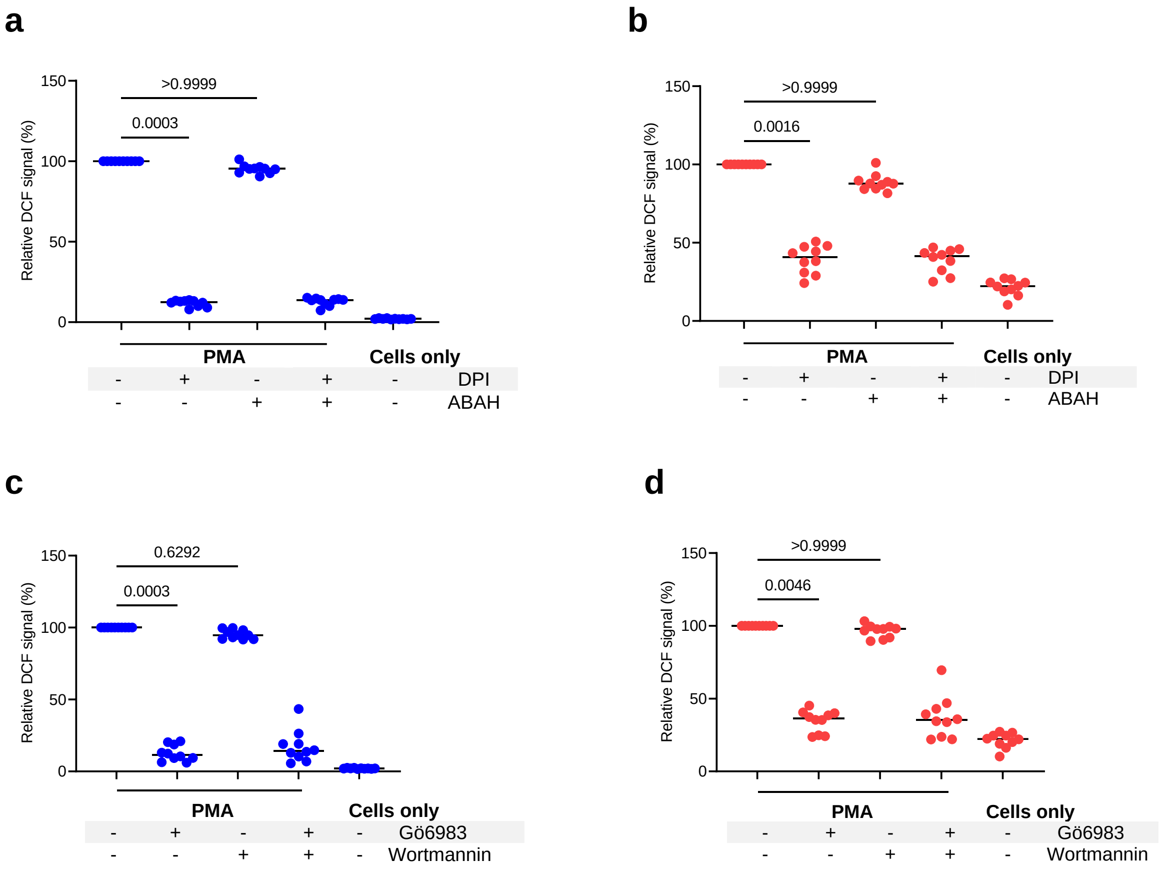

Supplementary Figure 2. PMA-induced production of reactive oxygen species depends on NADPH oxidase (NOX2) and the PKC signalling pathway.

Peripheral blood leukocytes (PBLs) were incubated with PMA (10 ng/ml) after pre-treatment with 2.5 µM diphenyleneiodonium (DPI, an inhibitor of NOX2) and/or 500 µM 4-aminobenzoic acid hydrazide (ABAH, an inhibitor of MPO), 250 nM Gö6983 (an inhibitor of protein kinase C) and/or 100 nM wortmannin (an inhibitor of phosphoinositide 3-kinase). Graphs show the relative DCF signal of neutrophils (blue) and monocytes (red), using PMA-induced untreated cells as reference. Horizontal lines represent the median. P values were determined by the Friedman test and Dunn's multiple comparisons test. Values shown are from single independent experiment (n=12).

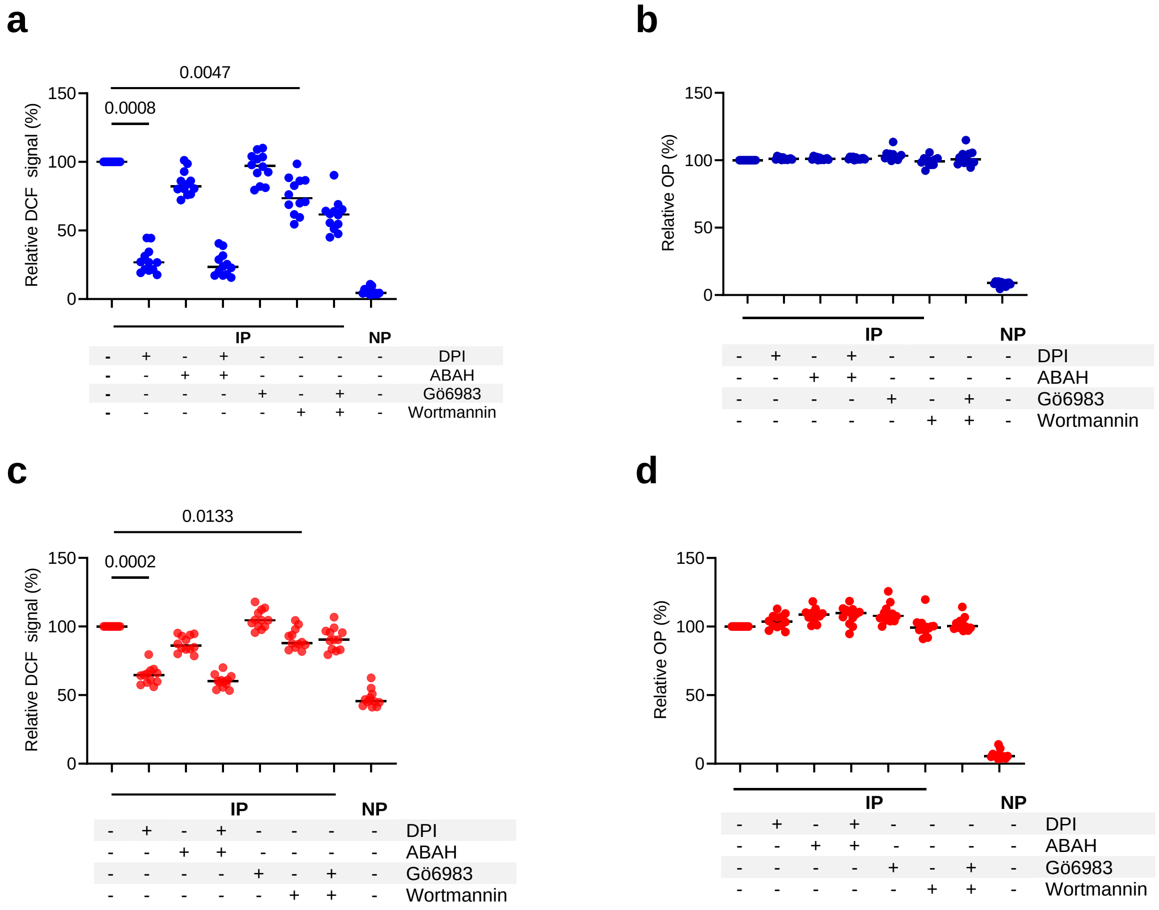

Supplementary Figure 3 Addition of enzyme inhibitors did not affect phagocytosis of opsonized merozoites.

Peripheral blood leukocytes (PBLs) were incubated with immune plasma (IP) or non-immune plasma (NP) plus 2.5 µM diphenyleneiodonium (DPI, an inhibitor of nicotinamide adenine dinucleotide phosphate (NADPH) oxidase (NOX2)), 500 µM 4-aminobenzoic acid hydrazide (ABAH, an inhibitor of myeloperoxidase (MPO)), 250 nM Gö6983 (an inhibitor of protein kinase C), and/or 100 nM wortmannin (an inhibitor of phosphoinositide 3-kinase) before the addition of opsonized merozoites. Graphs show the relative DCF or opsonic phagocytosis (OP) signal of neutrophils (a and b) and monocytes (c and d) using the untreated cells (IP) as reference. P values were determined by the Friedman test and Dunn's multiple comparisons test. Values shown are from single independent experiment (n=12).

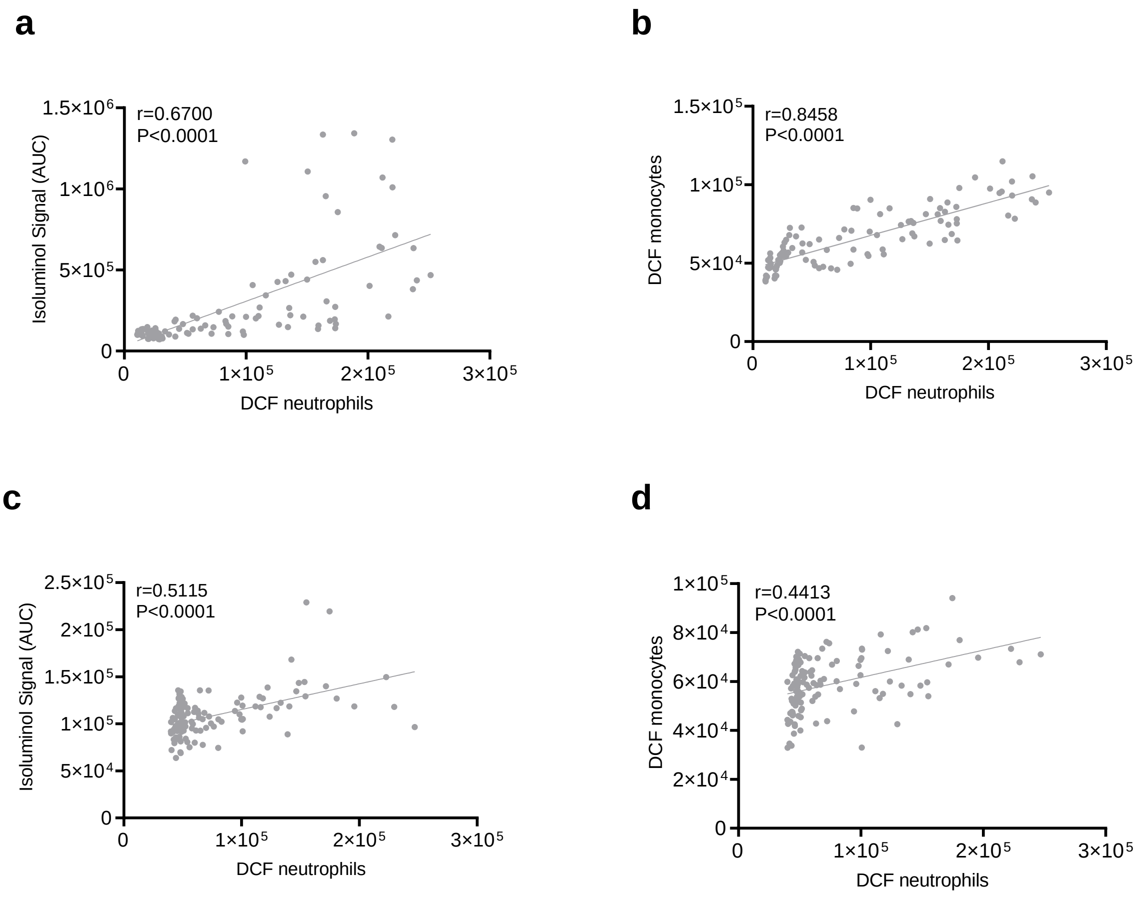

Supplementary Figure 4. Correlation between intracellular and extracellular reactive oxygen species (ROS) production of neutrophils and monocytes.

Scatterplots with linear regression lines show the relationship between intracellular and extracellular ROS production of neutrophils in Ghana a) and India c). Panels b) and d) depict the relationship between intracellular ROS production of neutrophils and monocytes in Ghana and India, respectively. The Pearson's correlation coefficient (r) and corresponding P values are shown in each plot.

Supplementary Table 1. Demographics of study participants and febrile malaria status during follow-up.

Parameter	Subgroup	Ghana	Subgroup	India
Total number		108		121
Age (years)	≤ 5	57 (52.8%)	≤ 10	48 (39.7%)
	≥ 6	51 (47.2%)	11-15	21 (17.4%)
			≥ 16	52 (43.0%)
Sex	Female	51 (47.2%)		59 (48.8%)
	Male	57 (52.8%)		62 (51.2%)
Bed net use	Yes	38 (35.2%)		14 (11.6%)
	No	70 (64.8%)		107 (88.4%)
Sickle cell trait	Negative	89 (82.4%)		Not available
	Positive	19 (17.6%)		Not available
Febrile malaria status	Susceptible	63 (58.3%)		48 (39.7%)
	Protected	45 (41.7%)		73 (60.3%)
