# Supplementary material for: Human blood neutrophils generate ROS through FcγR-signaling to mediate protection against febrile P. falciparum malaria
Source: Commun Biol. 2023 Jul 18;6:743. doi: 10.1038/s42003-023-05118-0 (PMC10354059; doi:10.1038/s42003-023-05118-0)
Supplement: Supplementary file 2 — Description of Additional Supplementary Files [file 42003_2023_5118_MOESM2_ESM.pdf]

Description of Additional Supplementary Files

File name: Supplementary Data
Description: The source Data behind figures 1, 2, 3, 4, 5 and 6.

File name: Supplementary Movie 1
Description: Phagocytosis and ROS production were assessed with the use of time-lapse confocal microscopy, related to Figure 1b. Purified neutrophils were incubated with ethidium bromide (EtBr)-stained merozoites opsonized with immune plasma (IP). EtBr-stained merozoites are shown in red and DCF signal in green. Images were taken every 4 seconds during a 4-minute timespan. The movie is sped up 20 times. The scale bar represents 10 µm.
