# Supplementary material for: Human blood neutrophils generate ROS through FcγR-signaling to mediate protection against febrile P. falciparum malaria
Source: Commun Biol. 2023 Jul 18;6:743. doi: 10.1038/s42003-023-05118-0 (PMC10354059; doi:10.1038/s42003-023-05118-0)
Supplement: Supplementary file 5 — Reporting Summary [file 42003_2023_5118_MOESM5_ESM.pdf]

nature portfolio

Corresponding author(s): Michael Theisen

Last updated by author(s): Jun 27, 2023

Reporting Summary

Nature Portfolio wishes to improve the reproducibility of the work that we publish. This form provides structure for consistency and transparency in reporting. For further information on Nature Portfolio policies, see our Editorial Policies and the Editorial Policy Checklist.

Statistics

For all statistical analyses, confirm that the following items are present in the figure legend, table legend, main text, or Methods section.

n/a	Confirmed	
☐	☒	The exact sample size (*n*) for each experimental group/condition, given as a discrete number and unit of measurement
☐	☒	A statement on whether measurements were taken from distinct samples or whether the same sample was measured repeatedly
☐	☒	The statistical test(s) used AND whether they are one- or two-sided<br>*Only common tests should be described solely by name; describe more complex techniques in the Methods section.*
☐	☒	A description of all covariates tested
☐	☒	A description of any assumptions or corrections, such as tests of normality and adjustment for multiple comparisons
☐	☒	A full description of the statistical parameters including central tendency (e.g. means) or other basic estimates (e.g. regression coefficient) AND variation (e.g. standard deviation) or associated estimates of uncertainty (e.g. confidence intervals)
☐	☒	For null hypothesis testing, the test statistic (e.g. *F*, *t*, *r*) with confidence intervals, effect sizes, degrees of freedom and *P* value noted<br>*Give P values as exact values whenever suitable.*
☒	☐	For Bayesian analysis, information on the choice of priors and Markov chain Monte Carlo settings
☒	☐	For hierarchical and complex designs, identification of the appropriate level for tests and full reporting of outcomes
☒	☐	Estimates of effect sizes (e.g. Cohen's *d*, Pearson's *r*), indicating how they were calculated

Our web collection on statistics for biologists contains articles on many of the points above.

Software and code

Policy information about availability of computer code

Data collection	No software was used.
Data analysis	R version 4.1.2 with the survival and lmtest packages<br>GraphPad Prism version 9

For manuscripts utilizing custom algorithms or software that are central to the research but not yet described in published literature, software must be made available to editors and reviewers. We strongly encourage code deposition in a community repository (e.g. GitHub). See the Nature Portfolio guidelines for submitting code & software for further information.

Data

Policy information about availability of data

All manuscripts must include a data availability statement. This statement should provide the following information, where applicable:

- Accession codes, unique identifiers, or web links for publicly available datasets
- A description of any restrictions on data availability
- For clinical datasets or third party data, please ensure that the statement adheres to our policy

The data generated in this study are provided in the Supplementary Data file. Data are also available from the corresponding authors upon request and pending agreement from relevant ethics committees for clinical data.

Human research participants

Policy information about studies involving human research participants and Sex and Gender in Research.

Reporting on sex and gender	Sex was self-reported.<br>Sex distribution of study participants is shown in Supplementary Table 1.<br>In the Ghanaian study, 51 (47.2%) participants were female and 57 (52.8%) were male.<br>In the Indian study, 59 participants (48.8%) were female and 62 (51.2%) were male.
Population characteristics	Demographics of study participants are described in Supplementary Table 1.<br><br>Age of Ghanaian participants (n=108) ranged from 1 to 12 years. Participants were divided into two groups: children 5 years and younger (52.8%) and children aged 6 and over (47.2%).<br>Individuals were categorized based on febrile malaria status during follow-up. Susceptible and protected individuals were 58.3 and 41.7% of the total.<br><br>In the Indian cohort (n=121) age ranged from 3 to 60 years. Participants were divided into three age groups: ≤10 years (39.7 %), 11 to 15 (17.4%), and ≥ 16 (43.0%)<br>Susceptible individuals represented 39.7% of the total and protected individuals 60.3%.
Recruitment	At enrollment, participants in both the Ghana and India were informed about the purpose of the study and an informed consent was obtained from study participants or their guardians before enrollment. The chance of any kind of bias which may influences the outcome of the results had been avoided by random inclusion of voluntary participants regardless of their gender, ethnicity, village, education, etc.<br>Samples from anonymous Danish blood donors (aged 18 to 60 years) were obtained at Copenhagen University Hospital. These individuals are residents of central Copenhagen and provided written consent to have a small portion of their blood stored anonymously and used for research purposes.
Ethics oversight	The Ghanaian longitudinal cohort study was approved by the Institutional Review Board of Noguchi Memorial Institute for Medical Research of the University of Ghana, Accra, Ghana.<br>The Indian longitudinal cohort study was approved by the Institutional Ethics Committee of the National Institute of Malaria Research, Indian Council of Medical Research, New Delhi, India.<br>Ethical approval for Danish blood donor samples was given by the Scientific Ethics Committee of Copenhagen and Frederiksberg, Denmark.

Note that full information on the approval of the study protocol must also be provided in the manuscript.

Field-specific reporting

Please select the one below that is the best fit for your research. If you are not sure, read the appropriate sections before making your selection.

☒ Life sciences ☐ Behavioural & social sciences ☐ Ecological, evolutionary & environmental sciences

For a reference copy of the document with all sections, see nature.com/documents/nr-reporting-summary-flat.pdf

Life sciences study design

All studies must disclose on these points even when the disclosure is negative.

Sample size	In total, the longitudinal cohort studies in Ghana and India included 798 and 945 individuals respectively. To avoid mislabeling non-exposed individuals as protected, only participants who were definitely exposed to Plasmodium falciparum were included in the analyses. Of these exposed individuals, plasma samples from 108 and 121 participants from the Ghanaian and Indian cohorts, respectively, were available.
Data exclusions	No data were excluded from the analyses.
Replication	The experimental findings presented in Figure 1 to 4 and Supplementary Figures 1 to 3 were from a single independent experiment. The number of independent assays is described in each figure legend.<br>The quantifications performed with cohort samples (Figures 5, 6 and Supplementary Figure 4) were not replicated due to limited sample availability.
Randomization	Randomization and blinding not applicable for these longitudinal cohort studies.
Blinding	Randomization and blinding not applicable for these longitudinal cohort studies.

Reporting for specific materials, systems and methods

We require information from authors about some types of materials, experimental systems and methods used in many studies. Here, indicate whether each material, system or method listed is relevant to your study. If you are not sure if a list item applies to your research, read the appropriate section before selecting a response.

Materials & experimental systems

n/a	Involved in the study
☐	☒ Antibodies
☒	☐ Eukaryotic cell lines
☒	☐ Palaeontology and archaeology
☒	☐ Animals and other organisms
☒	☐ Clinical data
☒	☐ Dual use research of concern

Methods

n/a	Involved in the study
☒	☐ ChIP-seq
☐	☒ Flow cytometry
☒	☐ MRI-based neuroimaging

Antibodies

Antibodies used	APC-conjugated anti-human CD45; clone HI30; BD Biosciences 555485; Lot 9291059<br>APC-AF750-conjugated antihuman CD14; clone TuK4; Thermo Fisher Scientific MHCD1427; Lot 2351088<br>BV421-conjugated anti-human CD66b; clone G10F5; BD Biosciences 562940; Lot 0314113<br>anti-human CD16; clone 3G8; BD Biosciences 555404; Lot 9290257<br>anti-human CD32; clone FLI8.26; BD Biosciences 555447; Lot 1179972<br>anti-human CD64; clone 10.1; BD Biosciences 555525; Lot 286490
Validation	Antibodies are routinely tested for their application for flow cytometry as stated in the manufacturer's website and the antibodies' technical data sheet. Each antibody has been used in previous studies and the relevant citations can be found in the manufacturer's website and the technical data sheet for each antibody.

Flow Cytometry

Plots

Confirm that:

☒ The axis labels state the marker and fluorochrome used (e.g. CD4-FITC).

☒ The axis scales are clearly visible. Include numbers along axes only for bottom left plot of group (a 'group' is an analysis of identical markers).

☐ All plots are contour plots with outliers or pseudocolor plots.

☒ A numerical value for number of cells or percentage (with statistics) is provided.

Methodology

Sample preparation	Peripheral blood leukocytes were isolated from blood samples of healthy Danish donors by centrifugation followed by red blood cell lysis. Cells were counted using a hemocytometer and distributed in 96-well U-bottom plates containing 5×10^4 cells in 100µl of cell medium per well. Then, 50µl of cell medium with 12µM of 2',7'-dichlorodihydrofluorescin diacetate (DCFH2-DA, 3µM final concentration) and 1:200 dilution of surface staining antibodies (1:800 final dilution) were added. The staining antibodies were BV421 anti-human CD66b (clone G10F5; BD Biosciences 562940), APC anti-human CD45 (clone HI30; BD Biosciences 555485), and APC-AF750 anti-human CD14 (clone TuK4; Thermo Fisher Scientific MHCD1427). Immediately, 50µl of merozoites resuspended in cell medium and opsonized with serum diluted 1:100 were added to each well. For phagocytosis assays, merozoites had been previously stained with 10µg/ml of ethidium bromide. After an incubation of 30 min. at 37°C, cells were washed thrice with FACS buffer (PBS with 0.5% BSA + 2mM EDTA). Samples were quantified with a CytoFLEX S (Beckman Coulter Life Sciences) flow cytometer
Instrument	CytoFLEX S; Beckman Coulter B75442
Software	Kaluza Analysis Software version 2.1
Cell population abundance	Whole blood leukocytes from whole blood were used without altering their natural abundance. Neutrophils and monocytes constituted approximately 60% and 6 % of all leukocytes respectively.
Gating strategy	Single events were gated by plotting FSC-H and FSC-A.<br>Then, leukocytes were gated based on positive CD45 signal.<br>Neutrophils were gated as CD66b+ and CD14- events in the leukocyte fraction.<br>Monocytes were gated as CD14+ and CD66b- events in the leukocyte fraction.<br>Phagocytic cells were gated based on ethidium bromide signal measured in the PE-CF594 channel (610/20 nm detector).<br>DCF signal was measured in the FITC channel (525/40 nm detector)

☒ Tick this box to confirm that a figure exemplifying the gating strategy is provided in the Supplementary Information.
